# Analgesic Effects of Low Dose Ketamine in Patients Undergoing Spine Fusion Surgery

**DOI:** 10.7759/cureus.113438

**Published:** 2026-07-27

**Authors:** Vaishali Singh, Bashir A Dar, Zoya Sehar, Prinka Devi, meenal sharma, Shehla Bashir

**Affiliations:** 1 Anaesthesiology, Sher-i-Kashmir Institute of Medical Sciences, Srinagar, IND

**Keywords:** intravenous patient-controlled analgesia, ketamine, non-opioid analgesia, pain severity, postoperative pain

## Abstract

Background: Postoperative analgesia in spine surgeries is necessary for postoperative recovery and wound healing. While opioids have their limitations, non-opioid analgesia is a growing field of interest. Ketamine, due to its analgesic properties, has emerged as a promising agent for postoperative analgesia. Our aim was to study the analgesic effect of low-dose ketamine infusion and to explore its potential for a multimodal pain management strategy.

Methods: This prospective observational cohort study was conducted at Sher-i-Kashmir Institute of Medical Sciences, a tertiary care hospital in Srinagar, India. A total of 44 patients were observed and divided into two cohort groups of 22 each based on their intraoperative analgesic regimen. One group had received a low-dose ketamine infusion (0.3 mg/kg/hour) both intraoperatively and postoperatively, and another group received regular analgesics (fentanyl, morphine, and paracetamol) except for the ketamine infusion. Pain score measured using the Visual Analog Scale (VAS), rescue analgesic requirement, and associated side effects were studied for a period of 48 hours.

Results: Ketamine administration led to a significantly lower VAS pain score at 4, 6, 12, and 18 hours postoperatively (p-value < 0.05). Adjustment for pair comparisons confirmed that the most substantial pain reduction occurred at four and six hours, indicating ketamine’s early-onset analgesic effect. Patients belonging to the ketamine group also demonstrated lower cumulative opioid use (mean difference: -16.00; CI: -19.68 to -12.32, P < 0.0001) at 48 hours postoperatively, higher satisfaction with pain management, and shorter hospital stays (mean: 4.45 days vs. 5.77 days), reinforcing ketamine's potential for improving early recovery outcomes.

Conclusion: Ketamine may demonstrate substantial promise as a safe and effective option for perioperative pain management, particularly in the early postoperative phase.

## Introduction

Spine fusion surgery is increasingly being performed worldwide as a definitive treatment for various spinal pathologies, including degenerative disc disease, spondylolisthesis, spinal deformities, and traumatic spinal injuries. Globally, the prevalence of spinal disorders and subsequent surgical interventions has risen significantly due to the aging population, sedentary lifestyles, and the increasing burden of degenerative musculoskeletal conditions. The annual growth rate of spine surgeries is estimated at 5% to 6%, driven by advancements in surgical techniques, better diagnostic tools, and an expanding patient pool requiring operative care [1]. Patients undergoing spine fusion procedures are at risk of persistent and acute postoperative pain. Also, they are at risk of developing opioid tolerance and postoperative hyperalgesia [2]. Effective pain control is, therefore, paramount in facilitating early mobilization, improving patient satisfaction, and enhancing overall clinical outcomes. Excessive use of opioids has been associated with adverse effects, including respiratory depression, nausea, vomiting, constipation, and the potential for opioid dependence and tolerance [3]. This growing awareness of the opioid crisis has prompted an exploration of alternative or adjunctive therapies to reduce opioid consumption and improve analgesic efficacy. Studies have demonstrated that low-dose ketamine not only enhances analgesia but also reduces opioid requirements and opioid-related side effects in various surgical populations, including those undergoing major orthopedic, abdominal, and thoracic surgeries [4, 5]. Its role in spine surgery, however, remains less well-defined, warranting further investigation. Loftus et al. demonstrated that intraoperative ketamine infusions reduced perioperative opioid consumption in opioid-tolerant patients undergoing spine surgery [6]. Similarly, Elia et al. conducted a systematic review and meta-analysis highlighting the efficacy of ketamine in treating postsurgical pain [7]. These findings underscore the potential benefits of incorporating ketamine into multi-modal analgesic protocols, particularly in high-risk populations. In conclusion, this study aims to bridge critical gaps in understanding low-dose ketamine’s role in spine fusion surgery.

We hypothesized that ketamine infusion used intraoperatively and postoperatively improves the pain scores, decreases opioid consumption, and improves the overall outcome of patients undergoing spine fusion surgery.

Our primary aim was to study the analgesic effect of an optimal low-dose infusion protocol of ketamine infused intraoperatively and postoperatively (0.3 mg/kg bolus followed by 0.3 mg/kg/hour infusion) in patients undergoing multi-segment spine fusion surgery and to explore its role as an analgesic in spine fusion surgery. Our secondary objectives were to study the cumulative opioid consumption in 48 hours and to study the adverse effects of ketamine infusion in the postoperative period.

## Materials and methods

Study design

This prospective observational cohort study was conducted at Sher-i-Kashmir Institute of Medical Sciences, a tertiary care hospital in Srinagar, India, from December 2023 to June 2025 for a period of 18 months.

Sample size calculation

A total of 44 patients divided into two cohort groups (Group A: ketamine and Group B: non-ketamine), with 22 patients in each cohort, were observed. The sample size was calculated based on a previous study estimating the primary outcome of postoperative Visual Analog Scale (VAS) pain scores. Assuming an expected mean difference (d) of 1.7 between the groups and a standard deviation (SD) of 2.0, a minimum sample size of 22 patients per group (total of 44 patients) was required to achieve a statistical power of 80% with a one-tailed significance level (α = 0.05).

Institutional Ethical Committee (IEC) clearance was obtained from the Sher-i-Kashmir Institute of Medical Sciences, Srinagar (IEC number 291/2023).

A Strengthening the Reporting of Observational Studies in Epidemiology (STROBE) checklist was used for this study.

Inclusion criteria

Patients aged 18-70 years of either gender, classified as American Society of Anesthesiologists (ASA) physical status I-III, scheduled to undergo multi-segment (≥2 levels) spine fusion surgery, and willing to provide consent for postoperative patient-controlled analgesia were included in the study.

Exclusion criteria

We excluded patients who refused to participate in the study, those with ASA physical status ≥ IV, those with poorly controlled blood pressure (BP >160/100 mmHg), and those with a history of hyperthyroidism, hypercholesterolemia, pheochromocytoma, schizophrenia, epilepsy, Parkinson's disease, sick sinus syndrome, atrioventricular block, severe cardiac dysfunction, hepatic dysfunction, renal insufficiency, or severe dementia.

Methodology

The analgesic protocol received by the patients was at the discretion of the attending anesthesiologist. In our study, we recruited patients undergoing spinal fixation surgery and noted their complete history and the analgesic protocol that they received. Based on that, they were divided into different groups: Group A (ketamine) and Group B (non-ketamine). All patients received the standard general anesthesia drugs (injection of propofol at 1.5-2.5 mg/kg IV, injection of fentanyl at 2 mcg/kg IV, and injection of rocuronium at 0.5 mg/kg IV). The ketamine group, in addition, received a bolus of 0.3 mg kg⁻¹ ketamine intravenously about 30 min before incision, which was followed by a continuous infusion at a rate of 0.3 mg kg⁻¹ h⁻¹ until one hour before the end of surgery. After surgery, patient-controlled analgesia was provided with ketamine 0.3 mg kg⁻¹ h⁻¹, dexmedetomidine 100 micrograms, and tramadol 100 mg, diluted with normal saline to 100 ml. The pump was programmed to deliver 2-ml boluses with a background infusion rate of 1 ml/h and a 10-min lockout interval. The said infusion was continued for 48 hours post surgery. Group B (non-ketamine), on the other hand, was not given any perioperative ketamine. After surgery, patient-controlled analgesia in this group was provided with dexmedetomidine 100 micrograms and tramadol 100 mg, diluted with normal saline to 100 ml. The pump was programmed similarly to the ketamine group to deliver 2-ml boluses with a background infusion rate of 1 ml h⁻¹ and a 10-min lockout interval. The said infusion was continued for 48 hours post surgery. The intraoperative need for an analgesic supplement for both groups was addressed using an injection of fentanyl 50 mcg bolus IV or an injection of morphine 3 mg IV after assessing nociceptive parameters like heart rate > 120 beats per minute (bpm) or systolic BP > 20% above baseline. The intraoperative additional opioid consumption was calculated and noted as morphine equivalents for both groups. Postoperatively, all patients received 1 g of paracetamol IV every eight hours. The pain scores were noted by the concerned nurse at various time points (two hours, four hours, six hours, 12 hours, 18 hours, 24 hours, and 48 hours), and in case of a VAS score ≥ 6, rescue analgesia was provided in the form of opioid morphine 3 mg IV or injection fentanyl 50 mcg IV. The opioids received postoperatively were noted and calculated as morphine equivalents for both groups. None of the patients in our study cohort received regional block for pain. While intraoperative and postoperative analgesic management was left to the discretion of the attending anesthesiologist, the institutional practice within our department follows a standardized multi-modal analgesia pathway. The primary analgesic priorities consist of scheduled paracetamol and nonsteroidal anti-inflammatory drugs combined with patient-controlled analgesia, regional nerve blocks, and rescue opioids as indicated by clinical indications.

Primary outcome measure

To compare the pain scores between the two groups within 48 hours post surgery, the time points at which pain scores were compared between the two groups were two hours, four hours, six hours, 12 hours, 18 hours, 24 hours, and 48 hours. 

Secondary outcome measures

To study opioid consumption during anesthesia from the induction to the end of anesthesia, with opioid usage quantified in equivalent doses of morphine. The cumulative opioid consumption after surgery was tracked from the end of anesthesia up to the second day post surgery. The length of hospital stay after surgery was monitored until discharge. Patients were interviewed at the time of discharge to complete the Surgical Satisfaction Questionnaire-8 (SSQ-8) [8]. Finally, the incidence of postoperative complications (delirium, hallucinations, anxiety, depression, and nightmares) and mortality was recorded up to two days after surgery.

Statistical analysis

The data were compiled and entered in a spreadsheet (Microsoft Excel) and then exported to the data editor of IBM SPSS Statistics software, version 28.0 (IBM Corp., Armonk, NY, USA). The Kolmogorov-Smirnov or Shapiro-Wilk test was applied for the normality test. A range of statistical methods was employed in this study to appropriately analyze data based on variable type, distribution, and study design. Continuous variables were presented as mean ± SD. Baseline characteristics and VAS scores at individual time points were compared between the ketamine and non-ketamine groups using an independent student’s t-test. To control the family-wise error rate across the seven postoperative time points, the Holm-Bonferroni correction was applied. Independent t-tests were used to compare continuous variables. Categorical variables were analyzed using the chi-square test. For variables with small sample sizes or expected cell counts below five, such as ASA classification and rescue analgesia use, Fisher’s exact test was applied due to its higher accuracy under such conditions. For skewed or non-normally distributed variables such as time to first ambulation, the non-parametric Mann-Whitney U test was used to compare medians between the groups.

## Results

The comparative analysis revealed no significant differences in baseline demographic and clinical variables between the ketamine and non-ketamine groups, including age, gender, and ASA classification, confirming the comparability of the cohorts (Table 1).

**Table 1:** Distribution of age, gender, ASA status across ketamine and non-ketamine group ASA: American Society of Anaesthesiology; CI: confidence interval; N: number of patients; %: percentage; SD: standard deviation; p-value < 0.05 as significant; * t-test; ** chi-square test; ***Fisher exact test.

Groups		Ketamine (n=22) Mean ± SD	Ketamine (n=22) CI (lower, upper)	Non-ketamine (n=22) Mean ± SD	Non-ketamine (n=22) CI (lower, upper)	p-value
Age(years)		42.27 ± 15.62	(35.74, 48.80)	42.32 ± 14.43	(35.92, 48.72)	0.992 *
Sex	Female	9(40.9)	(23.3,61.3)	11(50.0)	(30.7, 69.3)	0.545 **
	Male	13(59.1)	(38.7,76.7)	11(50.0)	(30.7, 69.3)	
ASA	I	8(36.4)	(19.7,57.0)	8(36.4)	(19.7, 57.0)	0.885 ***
	II	12(54.5)	(34.7, 73.1)	11(50.0)	(30.7, 69.3)	
	III	2(9.1)	(2.5,27.8)	3(13.6)	(4.7,33.3)	

**Figure 1:**
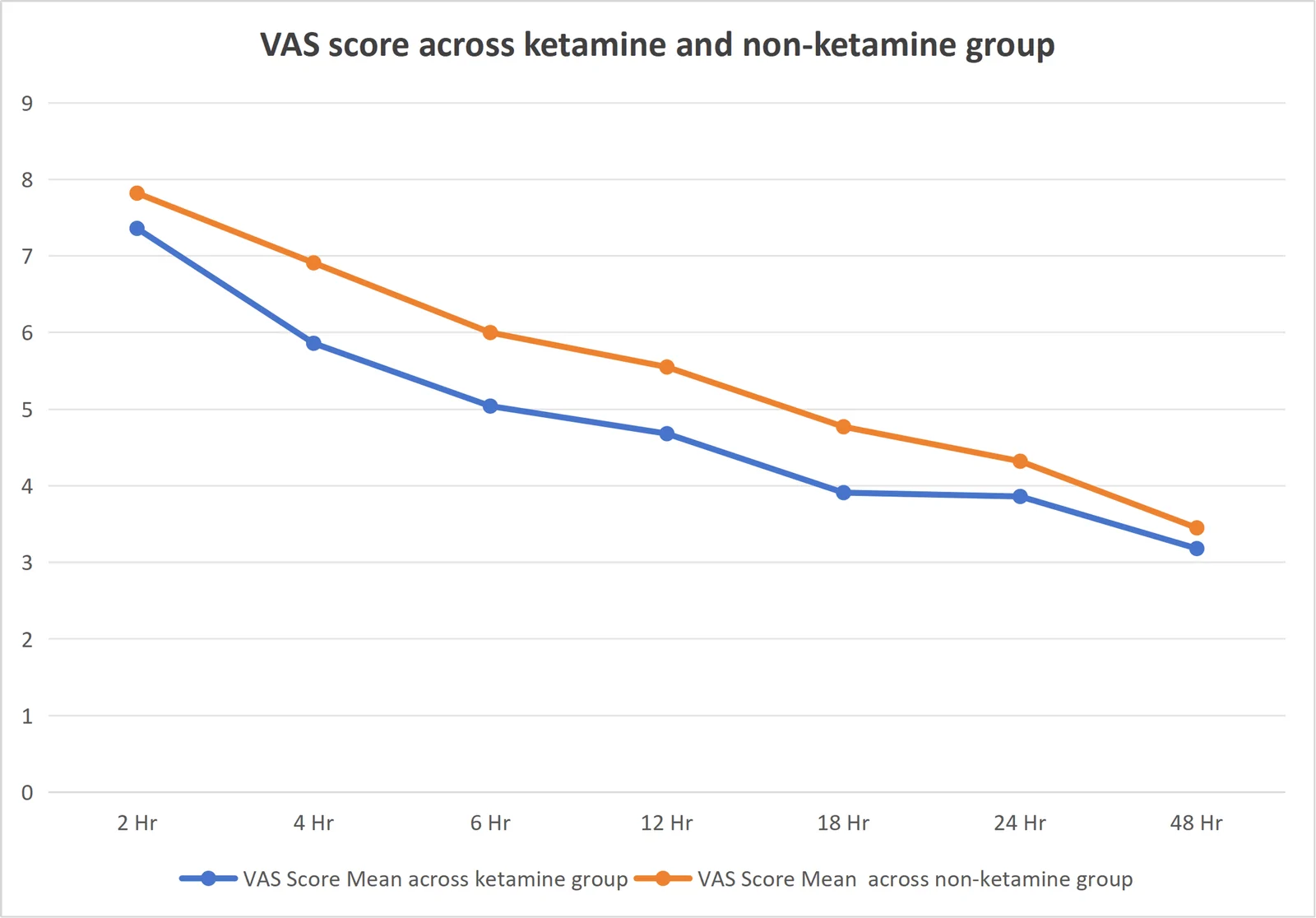
Mean VAS score across ketamine and non-ketamine groups VAS: Visual Analog Scale

The VAS for the ketamine group showed a significant reduction in pain over time, with the mean score decreasing from 7.36 at two hours to 3.18 at 48 hours. The standard deviations remained relatively low throughout, indicating a consistent pain response among participants. The VAS scores for pain in the non-ketamine group showed a consistent decline over time, indicating a progressive reduction in pain intensity. The mean VAS score for the non-ketamine group started at 7.82 at two hours and decreased steadily to 3.45 by 48 hours. On comparing the two groups, it was found that the mean VAS score in the ketamine group was lower than in the non-ketamine group at all time points (Figure 1).

The ketamine group consistently exhibited lower mean scores compared to the non-ketamine group across all time points. An independent t-test was used, and the results indicate that statistically significant differences were observed at four, six, 12, and 18 hours (p-values 0.002, 0.005, 0.030, and 0.034, respectively). Following adjustment for multiple comparisons using the Holm-Bonferroni method, the reduction in pain scores remained statistically significant only at four hours and six hours, while nominal differences were observed at 12 hours (p-0.030) and 18 hours (p-0.034); these did not meet the adjusted significance threshold after multiple testing correction. Overall, the data suggest a time-specific impact of ketamine, most prominent between four and six hours (Table 2).

**Table 2:** Comparison of VAS score between ketamine and non-ketamine group VAS: Visual Analog Scale; SD: standard deviation; CI: confidence interval; P value<0.05 as significant; T value using independent t test

Duration	Groups	Mean ± SD	95% CI (lower-upper)	p-value* (T value)
Two hours	Ketamine	7.36 ± 1.05	6.90-7.82	0.159 (1.42)
	Non-ketamine	7.82 ± 1.05	7.36-8.28	
Four hours	Ketamine	5.86 ± 0.83	5.49-6.23	0.002 (3.17)
	Non-ketamine	6.91 ± 1.23	6.37-7.45	
Six hours	Ketamine	5.04 ± 0.95	4.62-5.46	0.005 (2.87)
	Non-ketamine	6.00 ± 1.16	5.49-6.51	
12 hours	Ketamine	4.68 ± 0.78	4.33-5.03	0.030 (2.20)
	Non-ketamine	5.55 ± 1.63	4.83-6.27	
18 hours	Ketamine	3.91 ± 0.92	3.50-4.32	0.034 (2.15)
	Non-ketamine	4.77 ± 1.60	4.06-5.48	
24 hours	Ketamine	3.86 ± 0.89	3.47-4.25	0.154 (1.44)
	Non-ketamine	4.32 ± 1.17	3.80-4.84	
48 hours	Ketamine	3.18 ± 0.85	2.80-3.56	0.386 (0.87)
	Non-ketamine	3.45 ± 1.18	2.93-3.97	

**Figure 2:**
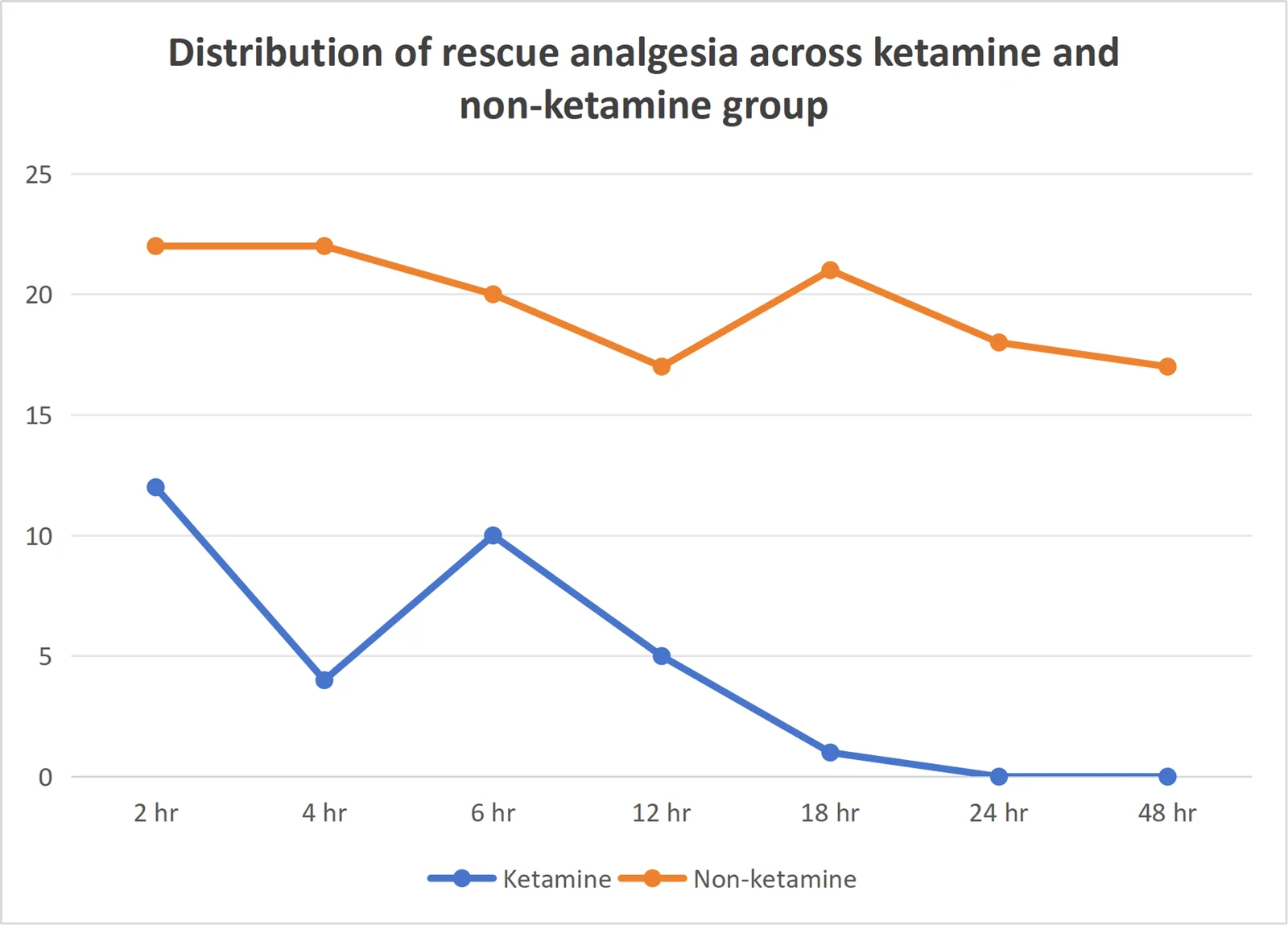
Distribution of rescue analgesia across ketamine and non-ketamine group hr: hour

A comparative analysis of the need for rescue analgesia (fentanyl, paracetamol, and tramadol) over time between patients receiving ketamine and those not receiving ketamine was done. At all measured time points two, four, six, 12, 18, 24, and 48 hours, the proportion of patients requiring rescue analgesia was significantly lower in the ketamine group compared to the non-ketamine group; Fisher's exact test was used with p-values consistently at 0.001, indicating strong statistical significance. Notably, at two and four hours, all patients in the non-ketamine group required rescue analgesia (22, or 100%), whereas only 12 (54.5%) and four (18.2%) of the ketamine group did so, respectively. By 24 and 48 hours, none of the ketamine group required rescue analgesia, in contrast to 18 (81.8%) and 17 (77.3%) in the non-ketamine group (Figure 2). 

The mean time to first ambulation was lower in the ketamine group (30.55 hours) compared to the non-ketamine group (37.68 hours), indicating a trend toward earlier mobilization with ketamine use. The Mann-Whitney U test was used, and the p-value of 0.242 indicated that this difference is not statistically significant. The comparison of hospital stay duration between the ketamine and non-ketamine groups showed a significantly shorter stay in the ketamine group (mean: 4.45 ± 1.26 days) compared to the non-ketamine group (mean: 5.77 ± 2.16 days). An independent t-test was used with a p-value of 0.018, suggesting there was a statistically significant difference. Additionally, overall satisfaction with pain management was significantly higher in the ketamine group by an independent t-test, reinforcing the potential benefit of ketamine in enhancing postoperative pain relief and patient comfort (Table 3).

**Table 3:** Comparison of various recovery metrics between ketamine and non ketamine group p-value < 0.05 significant; SD: standard deviation; SSQ: Surgical Satisfaction Questionnaire; df: degrees of freedom; *independent t test; #Mann-Whitney U test

Group		Mean ± SD	p-value*
Time to first ambulation (hours)	Ketamine	30.55 ± 15.15	0.242; #u value: 192; z score: -1.17
	Non-ketamine	37.68 ± 23.81	
Hospital stay duration(days)	Ketamine	4.45 ± 1.26	0.018; *t value: 2.48; df: 42
	Non-ketamine	5.77 ± 2.16	
Overall satisfaction using SSQ-8 questionnaire	Ketamine	6.59 ± 1.3	0.043; *t value: 2.08; df: 42
	Non-ketamine	5.91 ± 0.81	

There was no statistically significant difference in adverse events seen in both the ketamine and non-ketamine groups (Table 4).

**Table 4:** Comparison of various adverse events between ketamine and non-ketamine group N: number of patients; %: percentage of patients; p-value < 0.05 as significant; df: degrees of freedom; *Fischer's exact test; #-chi-square test

Adverse Event	Yes/No	Ketamine N (%)	Non-ketamine N (%)	p-value* (Fisher's exact test)
Delirium	Yes	0 (0.0%)	0 (0.0%)	1.0
	No	22 (100.0%)	22 (100.0%)	
Dizziness	Yes	7 (31.8%)	4(18.2%)	0.296; *(0.463)
	No	15 (68.2%)	18 (81.8%)	
Hallucination	Yes	2 (9.1%)	0 (0.0%)	0.147
	No	20 (90.9%)	22 (100.0%)	
Nightmares	Yes	3 (13.6%)	5 (22.7%)	0.435; *(0.147)
	No	19 (86.4%)	17 (77.3%)	
Mortality	Yes	0 (0.0%)	0 (0.0%)	1.0
	No	22 (100.0%)	22 (100.0%)	
Hospital anxiety and depression	Normal	6 (27.3%)	7 (31.8%)	0.932, # 0.155; df :2
	Borderline	7 (31.8%)	7 (31.8%)	

A cumulative opioid morphine equivalent consumption was seen to be statistically lower in the ketamine group in comparison to the non-ketamine group both intraoperatively and postoperatively at various time points (p < 0.001), thus emphasizing the opioid-sparing effect of ketamine (Table 5).

**Table 5:** Cumulative opioid consumption (morphine equivalents) Unpaired student t-test; p-value<0.05 as significant; n: number of patients

Cumulative morphine equivalents (mg)	Ketamine (n=22)	Non-ketamine (n=22)	Mean difference	T value	p-value	Confidence interval
Intraoperative	2 ± 1	10 ± 3	-8.00	11.87	<0.0001	-9.36 to -6.64
Four hours postoperative	4 ± 2	15 ± 7	-11.00	7.09	<0.0001	-14.13 to -7.87
12 hours postoperative	4 ±3	18 ± 8	-14.00	7.68	<0.0001	-17.68 to -10.32
24 hours postoperative	4 ± 3	20 ± 8	-16.00	8.78	<0.0001	-19.68 to -12.23
48 hours postoperative	4 ± 3	20 ± 8	-16.00	8.78	<0.0001	-19.68 to -12.32

**Figure 3:**
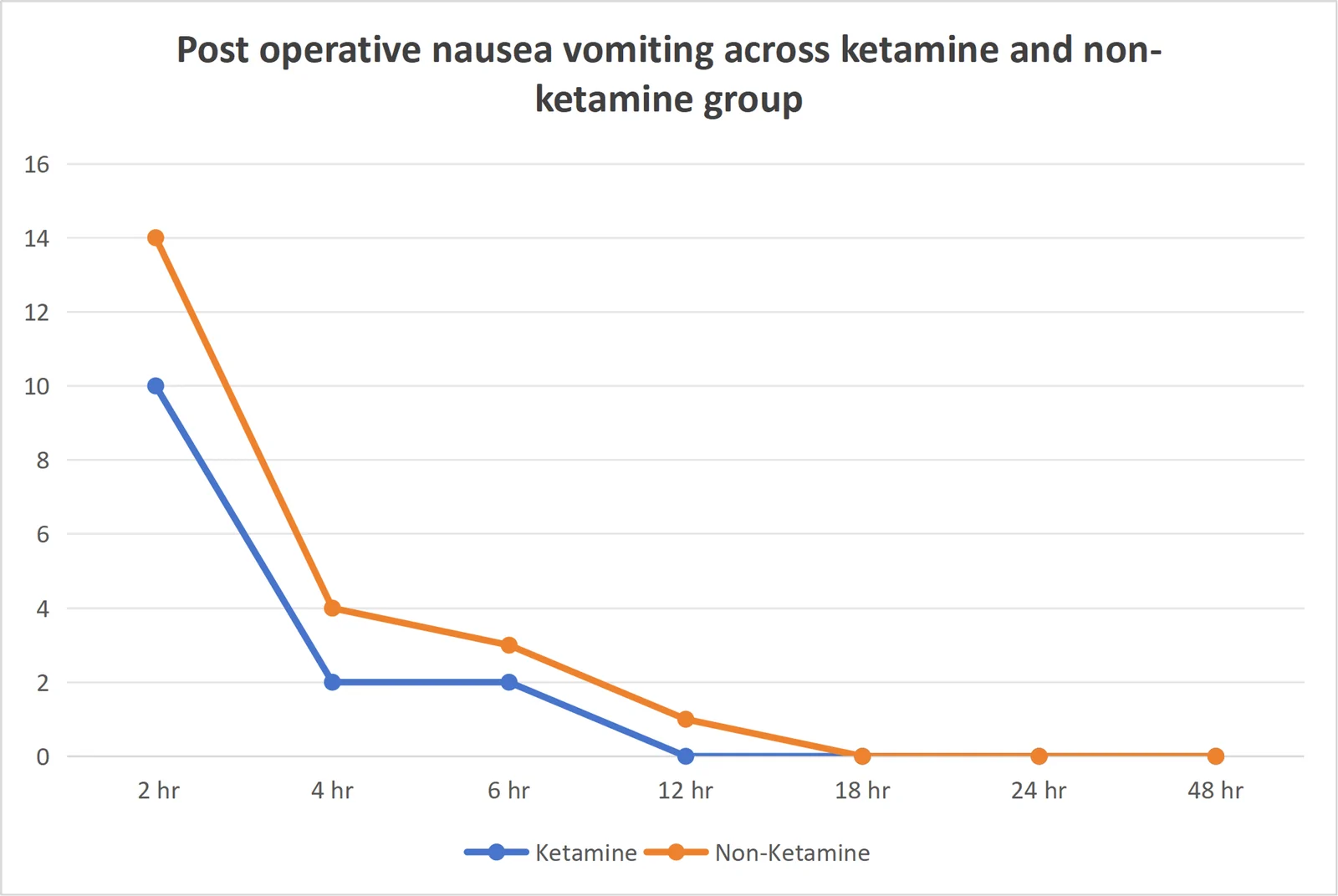
Postoperative nausea and vomiting across ketamine and non-ketamine groups hr: hour

The incidence of postoperative nausea and vomiting (PONV) decreased over time in both the ketamine and non-ketamine groups, with no cases reported after 18 hours. Although a higher percentage of PONV was observed in the non-ketamine group at two hours (63.6% vs. 45.5%) and four hours (18.2% vs. 9.1%), the differences were not statistically significant (p>0.05) at any time point by using the chi-square test (Figure 3).

This suggests that while there may be a trend toward lower PONV incidence in the ketamine group, the difference was not substantial enough to be considered statistically meaningful.

## Discussion

This study assessed the analgesic efficacy of ketamine in postoperative pain management by comparing pain scores, rescue analgesia requirements, and associated postoperative complications between patients receiving ketamine and those not receiving it. The analysis revealed clinically comparable groups, thereby minimizing the potential influence of confounding variables on analgesic outcomes. Pain assessment observed in our study (using the VAS) demonstrated a significant reduction in pain over time in both groups. Overall, the ketamine group consistently reported lower pain scores, with statistically significant differences observed at four hours (p = 0.002) and 6 hours (p = 0.005). These findings suggest that ketamine provides more effective pain control during the early postoperative period. At two hours, although pain scores were slightly lower in the ketamine group, the difference was not statistically significant (p = 0.159), indicating that the analgesic effect of ketamine may not be fully established immediately after surgery. By 24 and 48 hours, the differences were no longer statistically significant (p = 0.154 and p = 0.386, respectively), suggesting that ketamine's pain-relieving benefits are most prominent within the first 6 hours postoperatively. Additionally, the ketamine group generally exhibited lower standard deviations, indicating more consistent pain control. Clinically, these results highlight the value of ketamine as an adjunct in managing acute postoperative pain, particularly in the early recovery period, potentially leading to improved comfort, reduced opioid requirements, and faster functional recovery. A meta-analysis conducted by Tornøe et al. [9] on 2110 patients reported reduced 24-hour cumulative opioid consumption with a mean difference of −17.57 (95% CI −24.22 to −10.92, p < 0.01) at 24 hours postoperatively. Another meta-analysis conducted by Riddell et al. [10] found that patients receiving ketamine reported significantly reduced pain scores (VAS) at 24 and 48 hours with a decrease in total opioid use, which is consistent with our study. VAS was selected over the Numeric Rating Scale to reduce potential bias associated with numerical interpretation among individuals with limited literacy, allowing for a more accurate and inclusive assessment of pain.

The ketamine group in our study exhibited significantly lower rescue analgesia requirements (fentanyl, morphine) at all time points (p = 0.001) and a lower intraoperative and postoperative opioid (fentanyl, morphine) use (mean difference -16 at 48 hours). A statistically significant (p < 0.001) decrease in total opioid consumption was seen in the ketamine group at various time points (intraoperatively, postoperatively at four hours, 12 hours, 24 hours, and 48 hours). The finding supports the previous findings seen by Elia & Tramèr et al., who demonstrated that there was a significant decrease in cumulative morphine consumption at 24 hours postoperatively in patients with ketamine [7]. Similarly, Sharma et al. found that patients who received ketamine preemptively required lower doses of fentanyl in the postoperative period [11]. Park et al. emphasized ketamine’s efficacy in reducing opioid burden, particularly in surgeries associated with severe postoperative pain, such as spine surgeries, which showed that there was a significant decrease in the requirement of morphine and fentanyl in the postoperative period, which is in concordance with our study [12]. A systematic review by Riddell et al. evaluating low-dose ketamine in orthopedic surgeries found that patients receiving ketamine reported significantly reduced opioid consumption at 24 hours, supporting the findings of the present study [10].

Although the incidence of PONV was lower in the ketamine group, particularly within the first four hours postoperatively, the difference between the two groups was not statistically significant (p > 0.05). Some studies have reported ketamine's opioid-sparing effect as a contributing factor to reduced PONV; others have found no significant impact. Zhou et al., in a meta-analysis study, showed that the incidence of PONV was 27.8% in the ketamine group and 33.9% in the control group [13]. Early ambulation, decrease in duration of hospital stay, and overall improved satisfaction among patients in the ketamine group in the postoperative period, along with a decreased incidence of overall adverse events among them, signify the importance of using a non-opioid analgesic agent and suggest that ketamine use may be associated not only with improved pain management but also with enhanced recovery, potentially leading to earlier discharge. Liu et al. found that esketamine use was associated with a higher likelihood of discharge within three days post surgery, with an odds ratio of 2.28 (p = 0.014) [14]. Min et al. found that intraoperative administration of esketamine can facilitate earlier and more effective postoperative ambulation in elderly patients undergoing hip arthroplasty, which aligns with our study [15]. These findings highlight a potential role for ketamine in improving both patient satisfaction and clinical efficiency. Overall, these findings reinforce ketamine’s value as a safe and effective adjunct in postoperative pain management, with particular benefit during the early recovery phase, consistent with multiple prior studies and systematic reviews.

Limitations of the study

Some of the limitations of our study were a small sample size. Proper assessment could not be done in those who were paraplegic or quadriplegic. Patients were followed for VAS pain scores for a period of 48 hours only, and therefore, the role of ketamine in chronic pain management could not be studied. Also, preoperative use of analgesics in the form of opioids was not taken into consideration.

## Conclusions

The findings of this study suggest that ketamine enhances early postoperative pain relief and reduces opioid consumption. However, the analgesic advantage diminishes over time, indicating the necessity for adjunct pain management strategies beyond the immediate postoperative period. Considering our study limitations, we conclude that ketamine may demonstrate substantial promise as an effective option for perioperative pain management, particularly in the early postoperative phase.
